# Supplementary material for: First Description of the Nuclear and Mitochondrial Genomes and Associated Host Preference of Trichopoda pennipes, a Parasitoid of Nezara viridula
Source: Genes (Basel). 2023 May 27;14(6):1172. doi: 10.3390/genes14061172 (PMC10298747; doi:10.3390/genes14061172)
Supplement: Supplementary file 1 [file genes-14-01172-s001.zip › genes-2374351-supplementary.pdf]

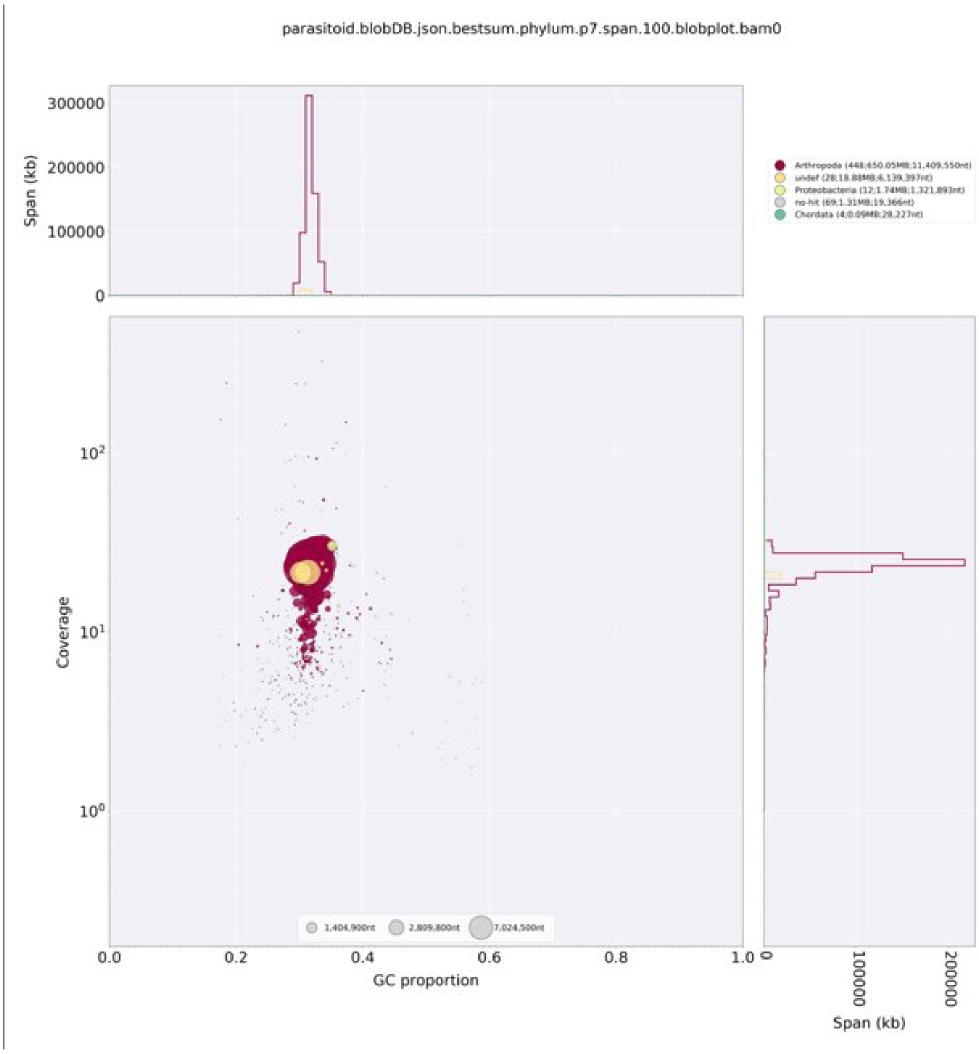

Figure S1. Blobplot (Taxon annotated GC-coverage scatter plot) of the contigs from the genome assembly. Each contig is plotted respective to their GC content and the depth of coverage. Each dot plot represents the contigs for the BLAST annotation with significant matches that are colored by putative taxon of origin.
